# Optimal microstructure and mechanical properties of open-cell porous titanium structures produced by selective laser melting

**DOI:** 10.3389/fbioe.2022.1022310

**Published:** 2022-10-04

**Authors:** Klaudia Kulcsár, Matej Buzgo, Pedro Ferreira Costa, Ibolya Zsoldos

**Affiliations:** ^1^ Department of Materials Science and Technology, Szechenyi Istvan University, Gyor, Hungary; ^2^ Dent-Art Teknik Ltd., Gyor, Hungary; ^3^ BIOFABICS LDA, Porto, Portugal

**Keywords:** porous microstructure, mechanical finite-element method (FEM), 3D pringting, computer tomograph, mechanical load test

## Abstract

Three-dimensional printing technology enables the production of open cell porous structures. This has advantages but not only in terms of weight reduction. In implant structures, the process of osseointegration is improved, mechanical integration is better, the open cell porous structures resemble a trabecular structure that mimics bone tissue. In this work, we investigated titanium structures made porous by cutting spheres. Based on the patterns of different types of crystal models we created porosity with different strategies. We have shown that there are significant differences in mechanical properties between the porous structures formed with different strategies. We determined the structure that loses the least load-bearing capacity compared to the solid structure, with the same porosity levels and mechanical stresses. We characterized the possibility location and environment of becoming an open cell structure. We performed the calculations with mechanical simulations, which were validated experimentally. The quality of the three-dimensional printing of samples was checked by computed tomography reconstruction analysis.

## 1 Introduction

It is estimated that 70%–80% of biomedical implants are made of metal (Li et al., 2014). Biomedical implants are used to replace hard tissues or bone, using commercially available pure titanium or titanium alloys (Wen et al., 2002). In recent decades, a significant design concept has been proposed for the design of bone tissue replacement structures considering mechanical properties, biological functionality, and biocompatibility (Goulet et al., 1994; Hutmacher, 2001). Recently, artificially porous, cellular lattice structures have been produced by additive manufacturing, as they are more like real bone structures, and their mechanical properties can also develop in a favourable direction (Van der Stok et al., 2013; Ahmadi et al., 2014; Amin Yavari et al., 2014). Additive manufacturing technologies have many advantages to produce porous structures because in this way more accurate and predictable structures can be created as opposed to traditional manufacturing processes (Mironov et al., 2009; Murr et al., 2010; Ahmadi et al., 2015; Chen et al., 2017a; Hatos et al., 2018).

In the 3D models of additive manufacturing, two different strategies have been applied for creating porosity. One strategy aims to build lattice structures with periodic, parallel shifts of different unit cells. In the case of the other strategy, the structures are made porous with a multitude of small cutting spheres. Both strategies make it easy and quick to build 3D CAD models, which can be perfectly used in additive technologies.

For the lattice structures, the structures constructed from different elementary cells were first compared (Ahmadi et al., 2015). The measurements were performed on the samples prepared by additive manufacturing, showing the maximum stresses, absorption energy and elasticity modulus. Later, the lattice structures optimized by mechanical properties were designed for titanium biomedical implants fabricated by additive technology (Du Plessis et al., 2018; El-Sayed et al., 2020; Zumofen et al., 2022). Trabecular and cubic topologies having various pore sizes were characterized and compared to each other (Hudák et al., 2021). Overviews of titanium lattice structures built from Schwartz primitive unit-cells were presented with different porosity levels (Soro et al., 2019a; Soro et al., 2019b; Attar et al., 2020).

The structure was made porous by a multitude of cut-out spheres, thus different open-cell structures with different porosities were studied (Chen et al., 2017b). The research investigated the geometric differences between the CAD models and the porous samples made with additive manufacturing, and SLM technology. It has been revealed how the mechanical properties may change with increasing porosity.

Recently, different papers have dealt with case studies on individual titanium dental (Rachmiel et al., 2017; Ramakrishnaiah et al., 2017), hip (Delikanli and Kayacan, 2019; Ghavidelnia et al., 2021) implants with porous structures produced by additive technologies, their placement, the osseointegration process (Anders et al., 2017), and finite element analyses (Kladovasilakis et al., 2020) The review articles on additive technologies provide separate chapters on (Cosma et al., 2018) or detailed descriptions (Krzysztof Pałka and Pokrowiecki, 2018) of porous implants. The advantages of porous structures are emphasized in more and more areas these days, e.g.: application to heat dissipating problems (Das and Sutradhar, 2020), grinding wheels (Li et al., 2021), tendon repair (Zhang et al., 2020), orthopaedics (Yu et al., 2020; Gao et al., 2021).

In this paper, we work with models of porous structures which are made porous by cutting spheres according to the 3D patterns of different crystalline structures. We show by mechanical finite element simulations that there are large differences in the mechanical properties of the samples made porous in different ways. Titanium samples are built from the samples with the best mechanical properties using additive technology. The simulation results are experimentally validated by pressure tests. The designed CAD models are compared with the CT reconstruction of the samples built with additive technology.

## 2 Models

The test specimens were designed based on the geometries of the most frequently occurring cubic–simple cubic, body-centred cubic, face-centred cubic–and diamond crystal structures. The 3D models were designed from crystal unit cells by cutting spheres out of solid unit-sized cubes in atomic locations. The radii of the spheres were gradually increased, thus reducing both the packing fraction and the mass of the structure. A mechanical analysis was conducted after each mass reduction step with finite element simulation. We determined the loadbearing capacity loss–in our case, compressive strength–of the structure after gradually reducing its packing fraction (mass). Manufacturability by 3D printing was also considered. This section presents the design of our 3D models. The results of finite element simulations will be discussed in Section 3.

In the first part of this study, 20 × 20 × 20 mm cubic unit bodies (unit cells) were used. Afterwards, these unit cells were packed together into 60 × 60 × 60 mm multiple-cell cubes containing 3 × 3 × 3 = 27 single unit cells, so that the mechanical analyses could be conducted on larger scale models.

### 2.1 Simple cubic structure

**FIGURE 1:**
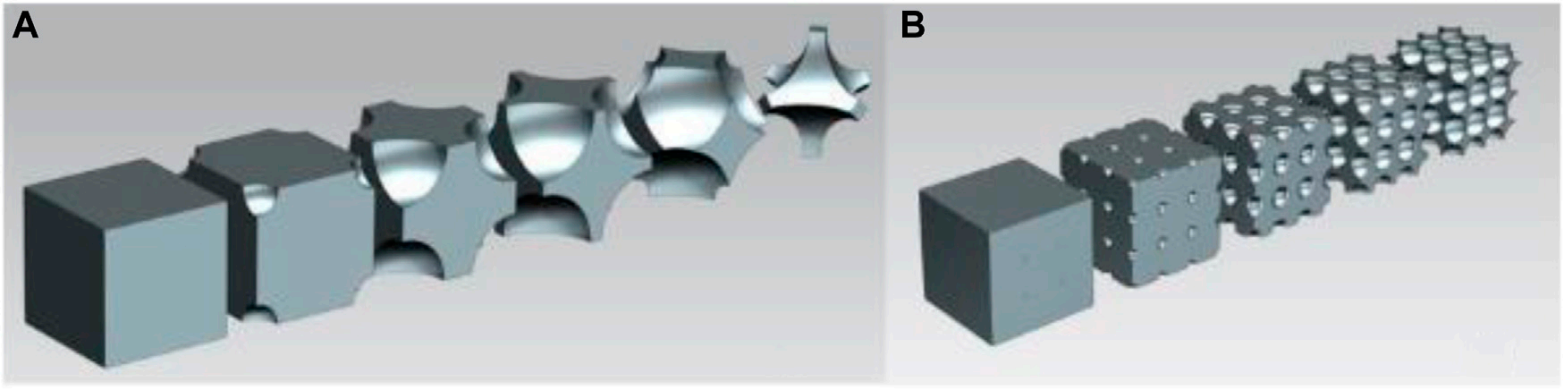
**(A)** Volume reduction of the unit cell in simple cubic structure; **(B)** Simple cubic structure geometry in multiple-cell model.

For the simple cubic structure, the cut-out spheres were in the corner points of the unit cube. Figure 1A shows the volume reduction of the single-cell simple cubic structure, and Figure 1B illustrates the geometry of the simple cubic cell structure in a multiple-cell volumetric model. The volume reduction was achieved by increasing the diameter of the cut-out spheres in 0.1 mm increments. This incremental increase applied to all cell types. As presented in the following figure, this geometry remained a closed-cell structure, which inhibited a large-scale volume reduction. Furthermore, the manufacturability of the structure by 3D printing was also questionable.

### 2.2 Face-centred cubic structure

**FIGURE 2:**
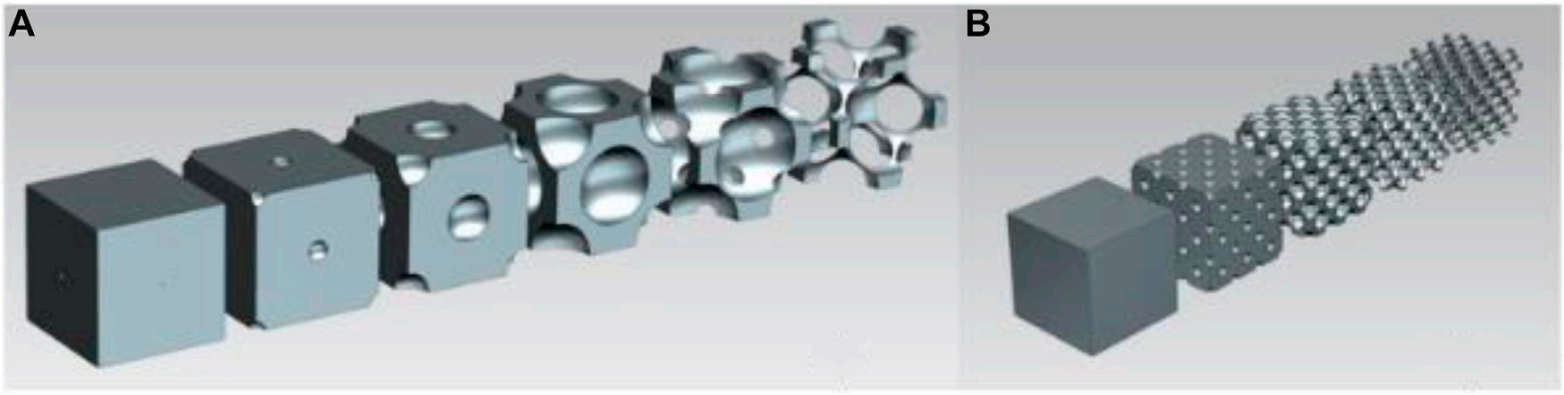
**(A)** Volume reduction of the unit cell in face-centred cubic structure; **(B)** Face-centred cubic structure geometry in multiple-cell mode.

For the face-centred cubic structure, the cut-out spheres were in the corner points and in the face centres of the unit cube. Figure 2A shows the volume reduction of the single-cell face-centred cubic structure, and Figure 2B demonstrates the geometry of the face-centred cubic cell structure in the multiple-cell volumetric model. As presented in the following figure, this geometry transitioned from a closed-cell to an open-cell structure, which enabled a large-scale volume reduction. Furthermore, 3D printing was a viable option to manufacture this design.

### 2.3 Body-centred cubic structure

**FIGURE 3:**
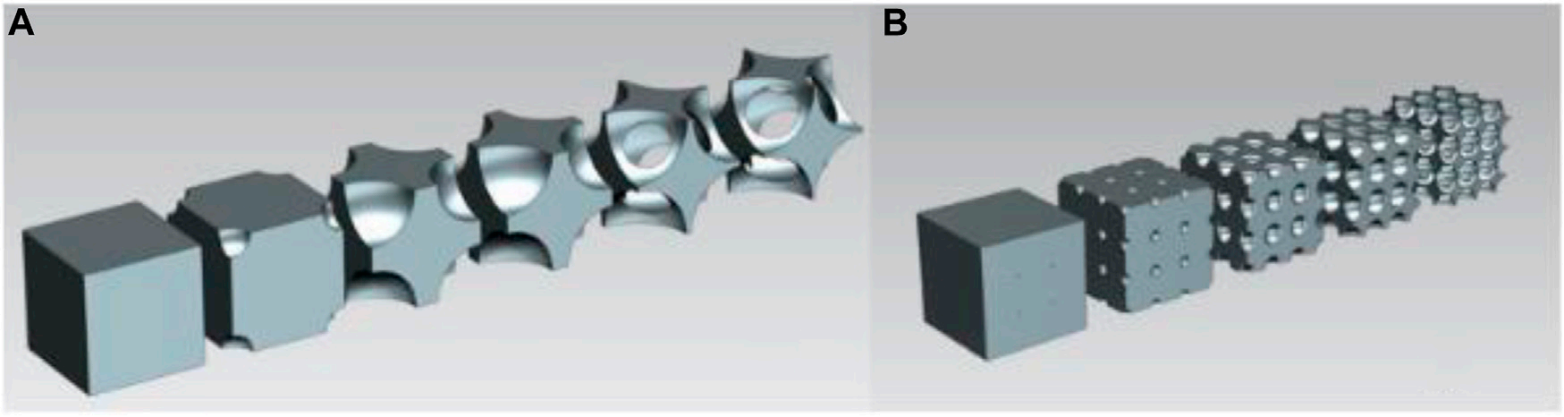
**(A)** Volume reduction of the unit cell in body-centred cubic structure; **(B)** Body-centred cubic structure geometry in multiple-cell model.

For the body-centred cubic structure, the cut-out spheres were in the corner points and in the body centre of the unit cube. Figure 3A shows the volume reduction of the single-cell body-centred cubic structure, and Figure 3B depicts the geometry of the body-centred cubic cell structure in the multiple-cell volumetric model. Like the face-centred cubic structure, this geometry also transitioned from a closed-cell to an open-cell structure, which enabled a large-scale volume reduction. Furthermore, 3D printing was also a viable option to manufacture this geometry.

### 2.4 Diamond cell structure

**FIGURE 4:**
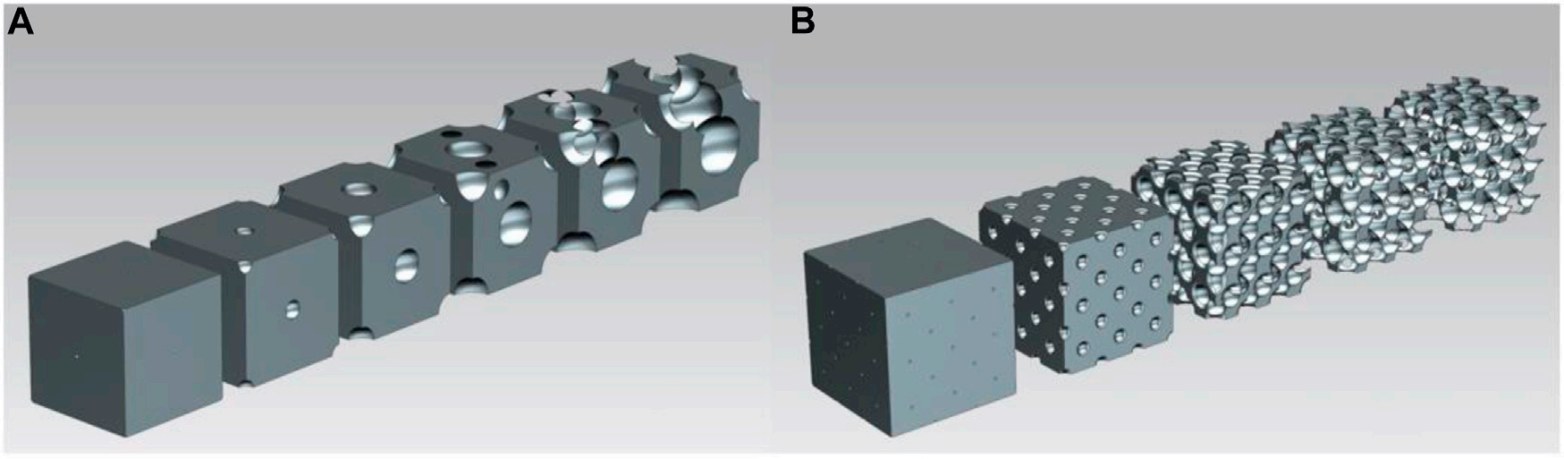
**(A)** Volume reduction of diamond cell structure; **(B)** Diamond cell structure geometry in multiple-cell model.

For the diamond cell structure, the cut-out spheres were in the corner points, in the face-centres, and in the centres of four opposite body octants. Figure 4A shows the volume reduction of the single-cell diamond structure, and Figure 4B illustrates the geometry of the multiple-cell lattice. Compared to the previous two cases, this geometry also transitioned from a closed-cell to an open-cell structure, which enabled a large-scale volume reduction. Furthermore, 3D printing was a viable option to manufacture this geometry.

## 3 Mechanical finite element analysis results

Ansys software was selected to run simulations with finite element analysis method. The initial generic cells of the single-cell structures were cubes of 20 × 20 × 20 mm for all the above-mentioned structure types. The multiple-cell models were made up of 3 × 3 × 3 = 27, thus unit cells add up to a total cubic volume of 60 × 60 × 60 mm. The volume reduction was achieved by increasing the diameter of the cut-out spheres in 0.1 mm increments until the integrity of the structure permitted. Permanent frictionless fixed constraints were applied on the bottom surface of the cubes. The applied load on the single-unit cell structures was 500 N. The load was increased in parallel to the surface increase of the multiple-unit structures i.e., the upper surface of 9 unit cells. Thus, we used 4500 N (9 × 500 N) load on the multiple-cell units. The mechanical stress levels were evaluated in multiple-unit structures considering the entire 27-unit body and the unit cube in the body centre. This enabled us to compare the behaviour of the separate single-cell models with that of the same unit cell embedded in continuous volume (i.e., when the unit cell was surrounded by the neighbouring unit cells from all directions). The stress equivalents in the overall body structures were calculated, which would be equal to the maximum stress value in the different locations of the volumetric model depending on its cell structure. “Sample data representative of Ti-6Al-4V, Additive Manufacturing” material model was used for the simulations and isotropic material model was utilised by the software accordingly for calculations.

### 3.1 Results for simple cubic models

**FIGURE 5:**
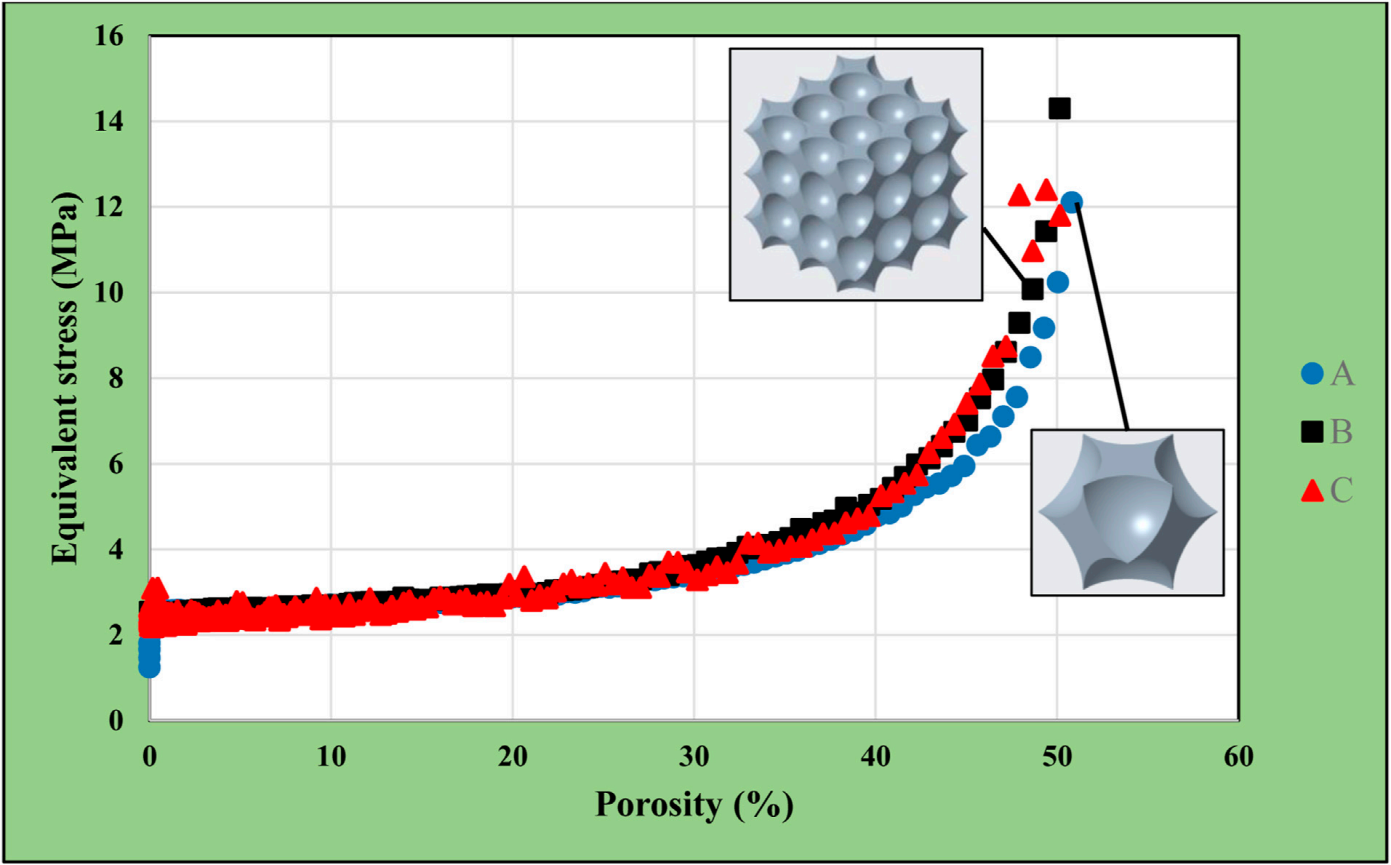
Equivalent stress as a function of porosity in single-cell and multi-cell structures of simple cubic alignment.

199 simulations were executed for single-unit and multiple-unit structures with simple-cubic alignment. The diameter was 0.1 mm for the smallest cut-out spheres, and 19.9 mm for the largest cut-out spheres. Figure 5 shows the comparative equivalent stresses in the single-cell and the multiple-cell models.

The diagram labels refer to the following information:

• Label “A” represents the equivalent stresses in the single-cell models with different porosities.
• Label “B” represents the equivalent stresses in the multiple-cell models with different porosities.
• Label “C” represents the equivalent stresses in the central unit cells of multiple-unit bodies with different porosities.

Porosity is defined as the volumetric ratio (in percentage) of the total volume of the cut-out spheres from the original (cubic) body.

As shown in Figure 5, simulations could only be executed up to the porosity of 51%. If the cut-out sphere size was increased further, the model collapsed with no continuous material volume left. The differences among the three cases (A, B, C) were not substantial, almost non-existent. This lattice design remained a closed-cell structure until final simulations.

### 3.2 Results for face-centred cubic models

**FIGURE 6:**
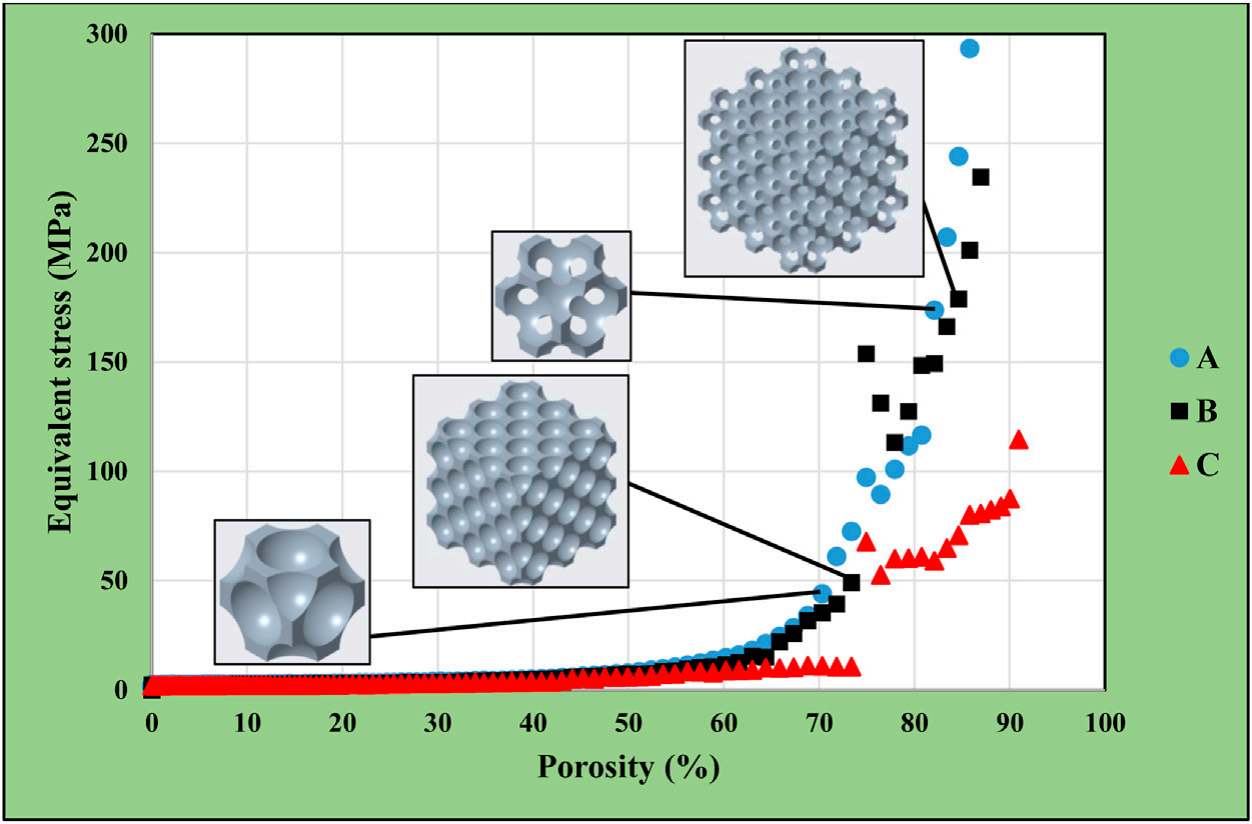
Equivalent stress as a function of porosity in simple single-cell and multi-cell structures of face-centred cubic alignment.

155 simulations were executed for both single-unit and multiple-unit structures with face-centred cubic alignments. The diameter was 0.1 mm for the smallest, and 15.5 mm for the largest cut-out spheres. Figure 6 shows the comparative equivalent stresses in the single-cell and the multiple-cell models. Labels on the diagram follow the previously defined logic:

• Label “A” represents the equivalent stresses in the single-cell models;
• Label “B” represents the equivalent stresses in the multiple-cell models;
• Label “C” represents the equivalent stresses in the central unit cells of the multiple-unit bodies.

It was noticeable in Figure 6 that the equivalent stress in the central unit cell of the multiple-unit body (curve C) was significantly lower than in the other two variations (curve A and B). This discrepancy was caused by the general structure of the body, as maximum mechanical stresses were located on the sides of the models. A high porosity could be achieved in the face-centred cubic models, and the stress results were still within the acceptable limit until 80% porosity.

**FIGURE 7:**
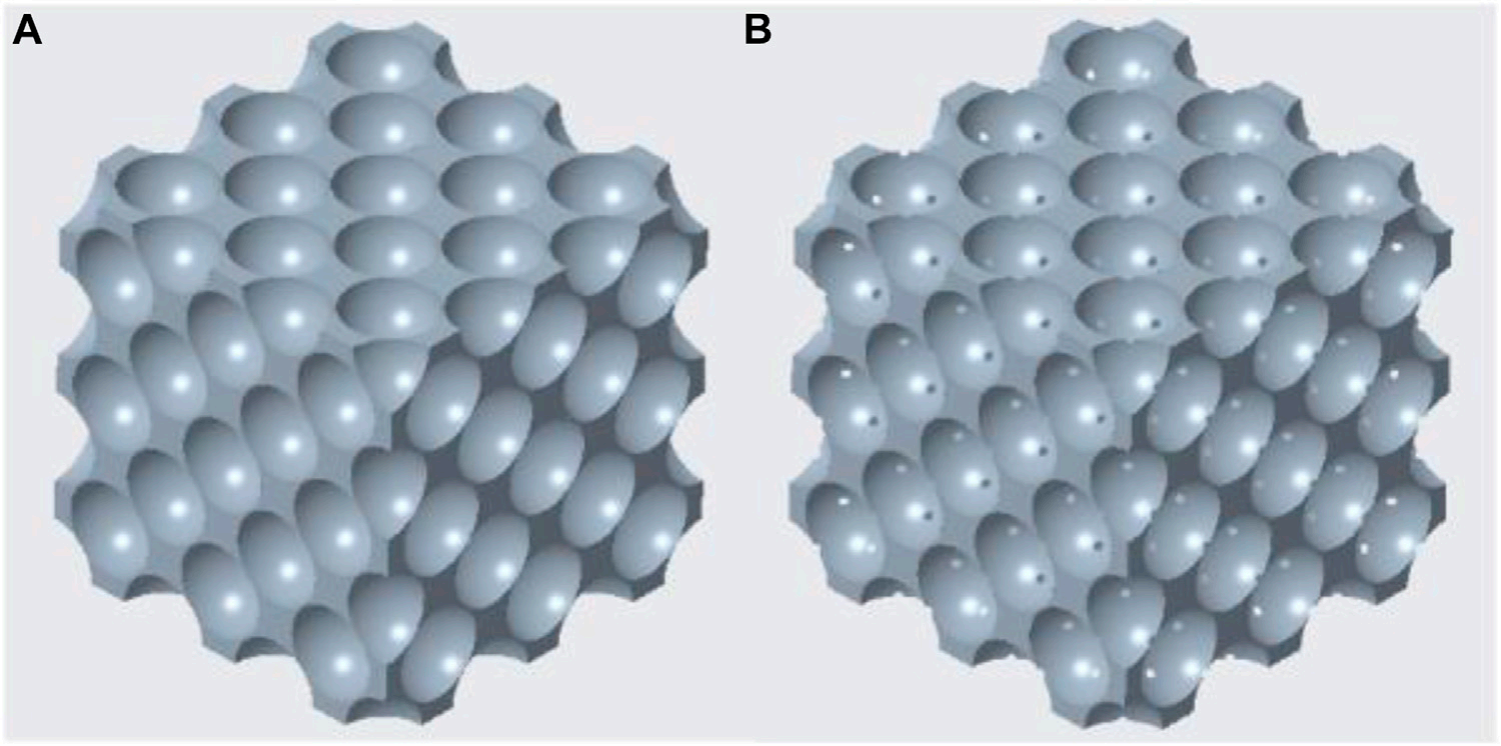
Transition of face-centred cubic model from closed-cell **(A)** to open-cell **(B)** lattices.

The curves had a breakpoint at 73%–74% porosity, after which a steep increase in the equivalent stress levels was noticeable. The reason for this was the transition from the close-cell to the open-cell structure at the porosity of 73%. This phenomenon is shown in Figure 7: the model remained a closed-cell lattice until 14.1 mm cut-out sphere diameter (Figure 7A); with 14.2 mm cut-out sphere diameter, the walls separating each pore were punctured and the model became an open-cell structure (Figure 7B).

**FIGURE 8:**
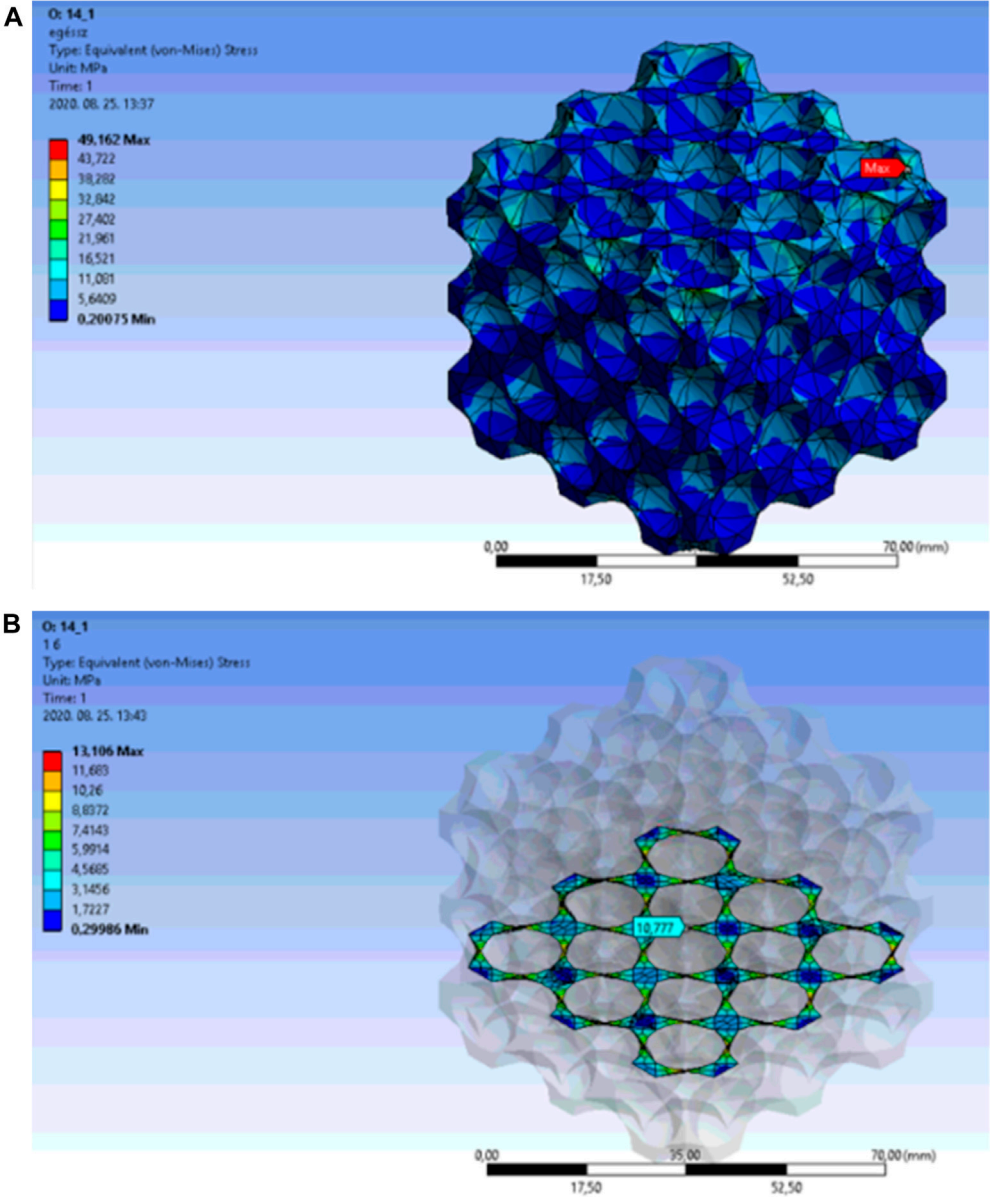
**(A)** Equivalent stress distribution in the overall lattice of face-centred cubic cell structure at a porosity of 73.388%. **(B)** Equivalent stress distribution in the internal area of the face-centred cubic cell structure at a porosity of 73.388%.

The finite element analysis results nearby the puncture point are presented in Figure 8. The porosity of the structure with 14.1 mm cut-out sphere diameter was 73.388%, for which case Figure 8A shows the equivalent stress distribution in the overall lattice structure. The red arrow marks the location of the maximum stress, which moved towards the side of the framework.

Figure 8B shows the maximum equivalent stress in the central unit cell on an appropriately selected section plane. The light blue arrow indicates the location of the peak stress on the internal edge of the unit cell.

**FIGURE 9:**
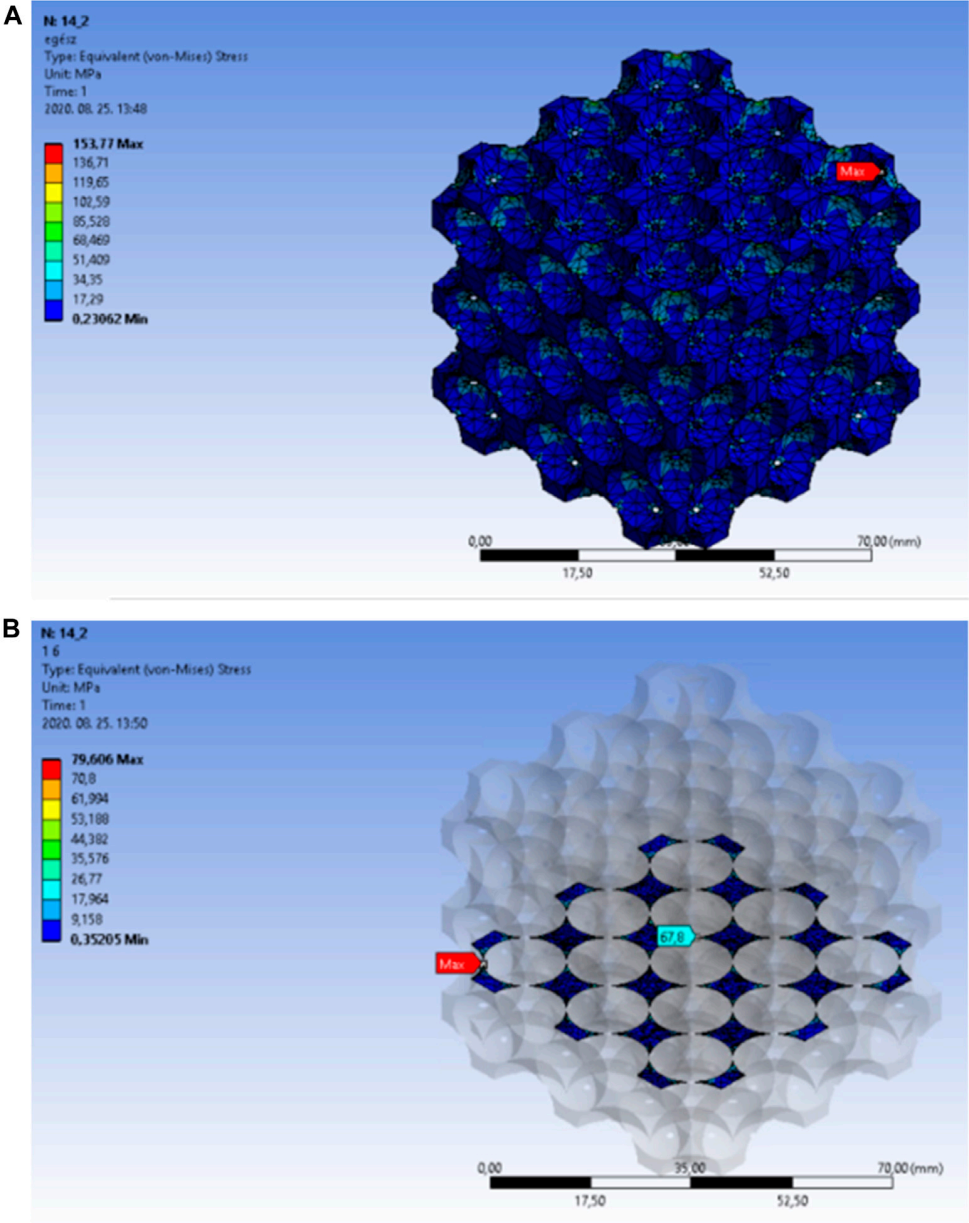
**(A)** Equivalent stress distribution in the overall lattice of face-centred cubic structure at a porosity of 74.94%. **(B)** Equivalent stress distribution in the internal area of the face-centred cubic cell structure at a porosity of 74.94%.

The locations of the peak stresses were also similarly distributed in the open-cell structures. The porosity of the structure with 14.2 mm cut-out sphere diameter was 74.94%, and the lattice transitioned to open-cell structure. The equivalent stress distribution in the overall body is presented in Figure 9A. The red arrow marks the location of the peak stress, which also moved towards the side of the framework.

Figure 9B shows the maximum equivalent stress in the central unit cell with 74.94% porosity on an appropriately selected section plane. The light blue arrow indicates the location of the peak stress on the internal edge of the unit cell.

### 3.3 Results for body-centred cubic models

**FIGURE 10:**
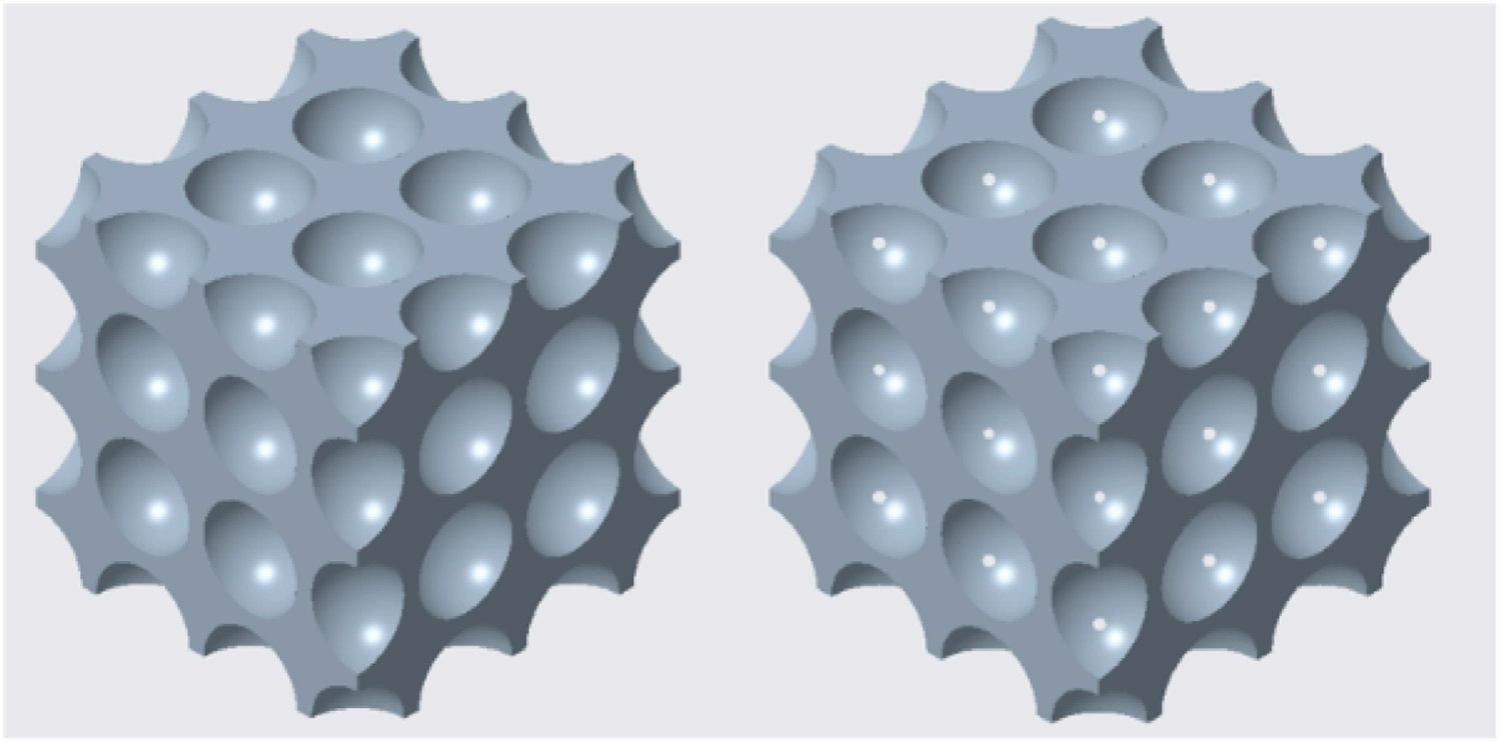
Equivalent stress as a function of porosity in simple single-cell and multi-cell structures of body-centred cubic alignment.

199 simulations were executed for both single-unit and multiple-unit structures with body-centred cubic alignment. The diameter was 0.1 mm for the smallest, and 19.9 mm for the largest cut-out spheres. Figure 12 shows the comparative equivalent stresses in single-cell and multiple-cell models. The labels on the diagram follow the previously introduced definitions. Figure 10 shows the transition of the model from the close-cell to the open-cell structure at the porosity of approximately 68%. This porosity value causes the breakpoint in curves, as well. The slope of the equivalent stresses steeply increased moving right from the curve breakpoint. All the three curves showed similar properties without any significant differences. The body-centred cubic structure presented the best results, as the equivalent stresses remained at acceptable levels up to the porosity of 92%.

The equivalent stress levels in the single-cell and the multiple-cell bodies with body-centred cubic structure showed a steep increase after reaching a porosity of 67%. This porosity value resulted in breakpoints in all curves. In this case, the breakpoints indicated the transition of the models from the closed-cell to the open-cell structures. The finite element simulation results at the breakpoints are presented similarly to the previous part.

**FIGURE 11:**
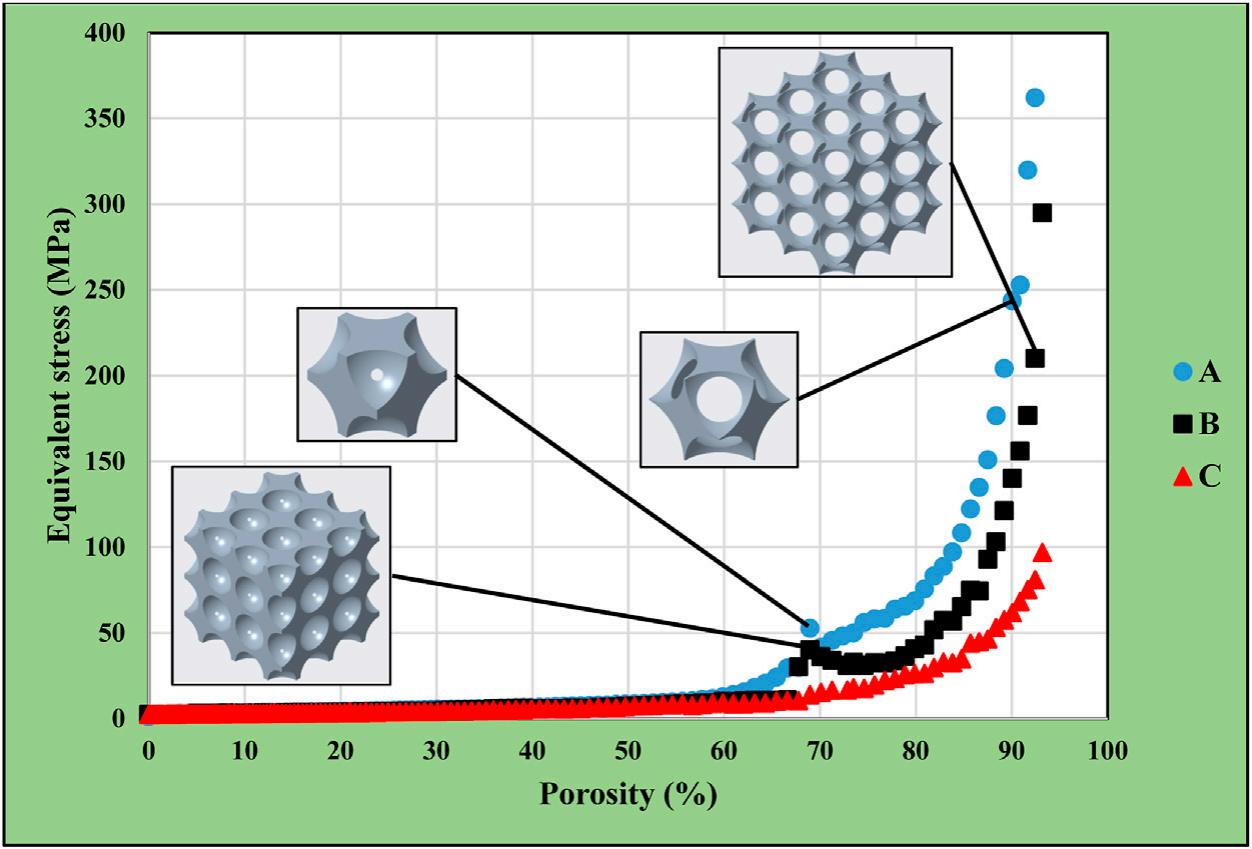
Equivalent stress as a function of porosity in simple single-cell and multi-cell structures of body-centred cubic alignment.

With a cut-out sphere diameter of 17.3 mm, the model remained a closed-cell structure. Afterwards, with a cut-out sphere diameter of 17.4 mm, the cell structure opened. The 3D models of this transition are presented in Figure 11.

**FIGURE 12:**
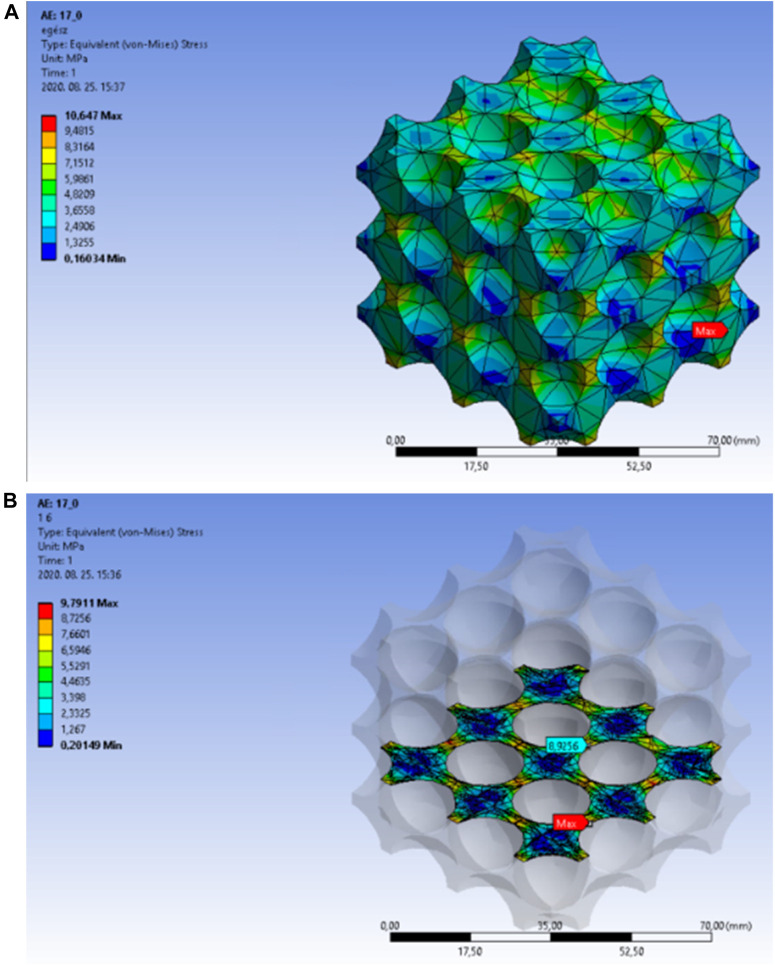
**(A)** Equivalent stress distribution in the overall body of the body-centred cubic cell structure at a porosity of 64.31%. **(B)** Equivalent stress distribution in the internal area of the body-centred cubic cell structure with a porosity of 64.31%.

In the next sections, the stress distribution results will be presented for the cell structure. 67.31% porosity could be achieved in the model that was created with a 17.3 mm cut-out sphere diameter. Figure 12A presents the equivalent stress distribution in the overall body. The red arrow marks the location of the peak stress, which moved towards the side of the structure this time, as well.

Based on the previously presented figures, Figure 12B shows the maximum equivalent stress in the central unit cell on an appropriately selected section plane. The light blue arrow indicates the location of the peak stress, which is predictively acting at the thinnest wall thickness.

**FIGURE 13:**
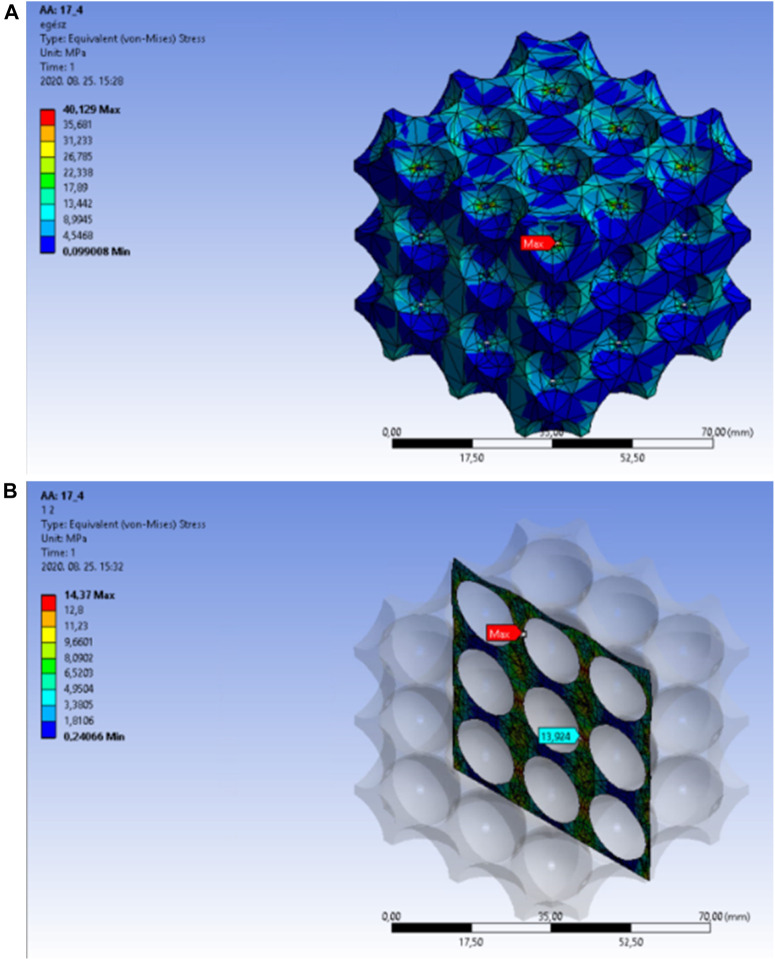
**(A)** Equivalent stress distribution in the overall lattice of body-centred cubic structure with a porosity of 68.95%. **(B)** Equivalent stress distribution in the internal area of the body-centred cubic cell structure with a porosity of 68.95%.

A porosity of 68.95% could be achieved by using 17.4 mm cut-out sphere diameters in the model. Figure 13A shows the equivalent stress distribution in the entire model. The red arrow indicates the location of the peak stress, which moved towards the side of the structure this time, as well.

In Figure 13B, the light blue arrow indicates the peak stress location in the central unit cell. The peak stress also emerged at the smallest wall thickness in this case, as well.

### 3.4 Results for diamond-cell-based models

**FIGURE 14:**
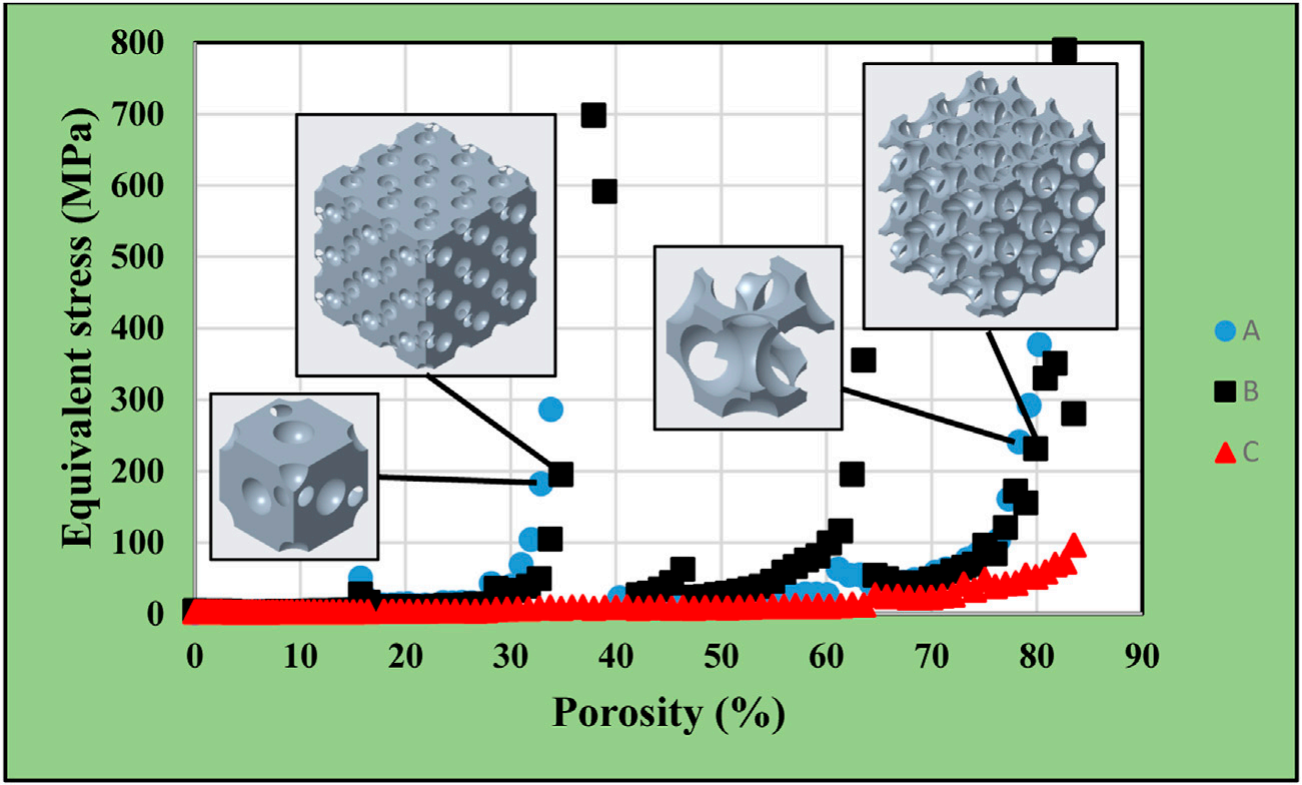
Equivalent stress as a function of porosity in simple single-cell and multi-cell structures of diamond-based alignment.

132 simulations were executed each for single-cell and multiple-cell bodies with a diamond cell structure. The diameter of the smallest cut-out sphere was 0.1 mm, and that of the largest one was 13.3 mm. Figure 18 shows the equivalent stress comparison between the single-cell and the multiple-cell models. We used the labels with the previously defined methodology. In this model type, many surface breakthrough point emerged increasing the cut-out sphere diameter. These breakthrough points (transitioning from a closed-cell to an open-cell structure) could be identified in the diagram with sudden sharp increases, as the stress levels in the thinning cross sections peaked just before achieving breakthroughs (Figure 14). The first sharp increase was noticed at a porosity of 15.74%, where the model first transitioned to an open-cell structure. Further sudden stress increases (cross sectional breakthroughs) were identified at 31%, 46%, and 61% porosities. The final results showed still acceptable stress level at the porosity of approximately 78%.

**FIGURE 15:**
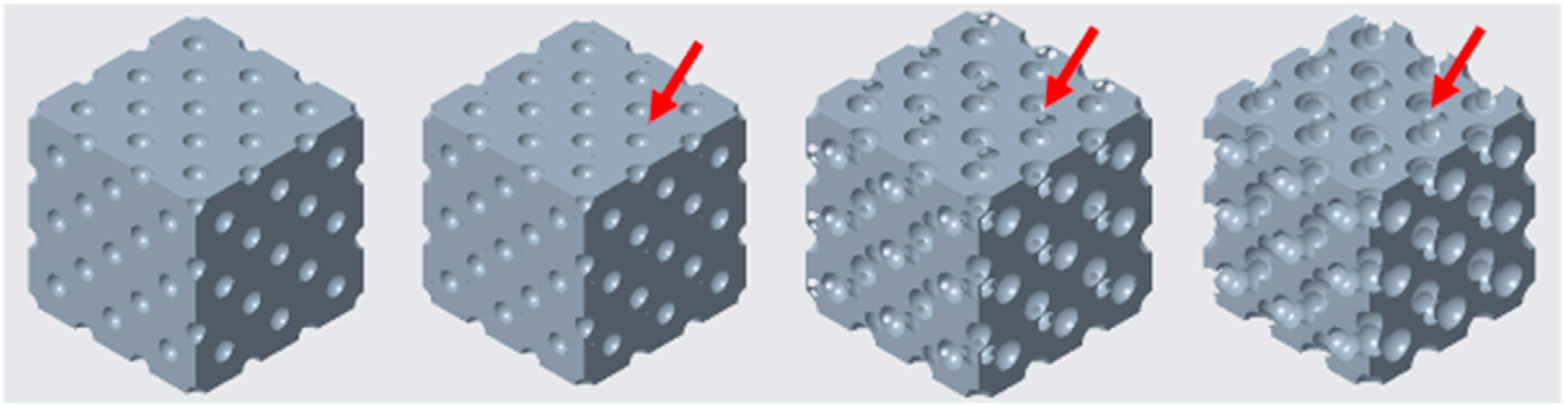
Closed-cell and open-cell structure of diamond-based alignment (from left to right 15.74%, 31%, 59.7% and 76% porosities).

Figure 15 presents the models after the different stages of cross-sectional breakthroughs. The red arrows indicate the locations of breakthrough points.

**FIGURE 16:**
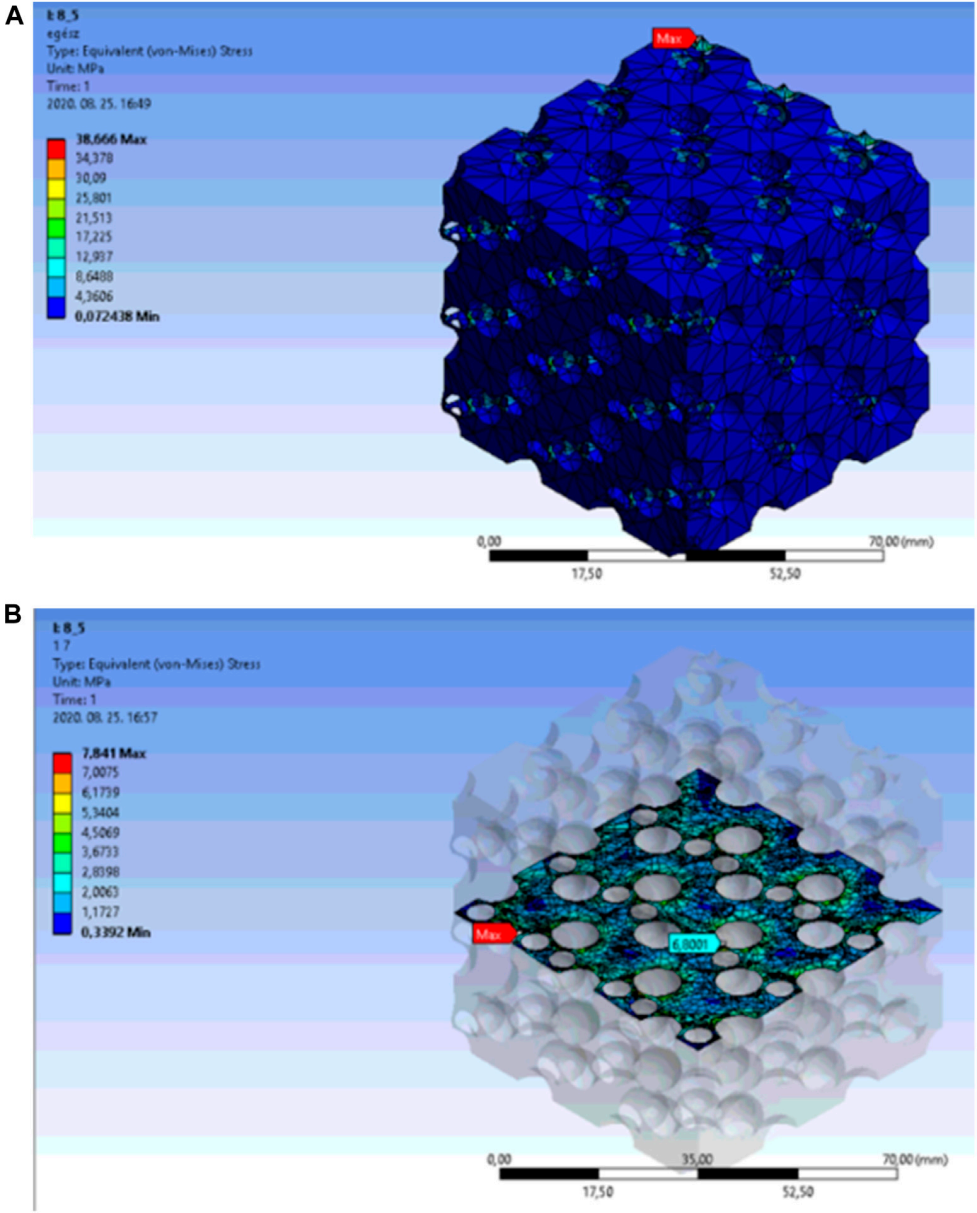
**(A)** Equivalent stress distribution in diamond lattice model at a porosity of 31.7%. **(B)** Equivalent stress distribution in the central unit cell of the diamond-based lattice at a porosity of 31.7%.

As an example, the finite element simulation results are presented for the second cross-sectional breakthrough at a porosity of 31.7%. In this case, the respective cut-out sphere diameter was 8.5 mm. Figure 16A Shows the equivalent stress distribution in the overall body. The red arrow indicates the location of the maximum stress, which emerged at the external side of the model.

Figure 16B. Presents the highest peak stress with the light blue arrow in the central unit cell with an appropriately selected cross-sectional plane. The location of the maximum stress just before the breakthrough was in the centre of the wall.

### 3.5 Comparison of results for different models

**FIGURE 17:**
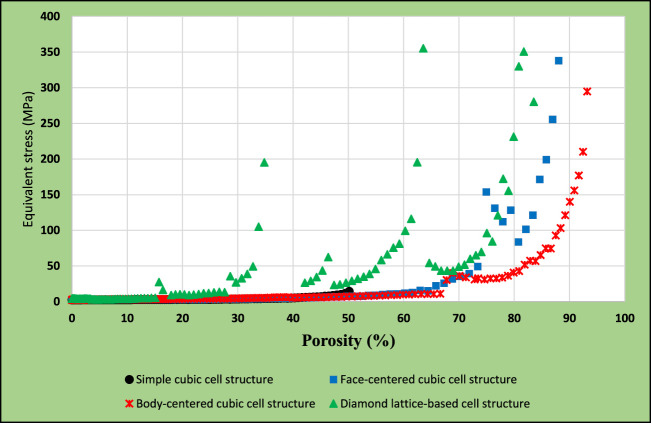
Equivalent stress comparison in different models.

The previously presented results were consolidated in Figure 17. This graph shows the maximum equivalent stresses in the simple cubic, face-centred cubic, body-centred cubic, and diamond based multiple-cell structures according to the function of porosity. The lowest volume reduction could be achieved in the simple cubic structure, which caused the lowest maximum equivalent stress compared to other designs. However, it would not be practical to use this structural alignment, as the model remained a closed-cell structure. The diagram shows 70–85% achievable porosity for the diamond-based lattice structure with substantially higher stress levels. For the face-centred cubic structure, a porosity of 80–85% just achieved the acceptable stress levels. The body-centred cubic structure resulted in an optimal solution as acceptable stress levels were achieved for up to 90–92% porosity during simulations.

## 4.3D printing of titanium specimens and CT reconstruction

The body-centred cubic lattice provided the best results from the previously described simulations. Three different test specimens were selected from the body-centred cubic structures. Printing of specimens was carried out with a SISMA Mysint 100 3D printer using laser beam melting. Throughout the process, the layer thickness, scan speed, and laser power were kept constant at 20 μm, 1,000 mm/s, and 125 W, respectively. For shielding, pure argon gas was used with a flow rate of 35 L/min. The specimens were subjected to CT reconstruction following their preparation with 3D printing. All the three selected models had open-cell structures that were suitable for 3D printing (the powder could be removed from the voids), and the calculations verified that the maximum stress levels remained within the acceptable limits for the compression tests. Ti-6Al-4V titanium powder was chosen for 3D printing. Table 1 shows the diameters of the cut-out spheres from the 3D model, which were measured during the CT reconstruction.

**TABLE 1:** Differences between 3D model and CT reconstruction.

	Diameter measured on the model	Diameter measured on CT reconstruction
Specimen no. 1	Φ 6.0 mm	Φ 5.85 mm
Specimen no. 2	Φ 6.2 mm	Φ 6.05 mm
Specimen no. 3	Φ 6.3 mm	Φ 6.16 mm

**FIGURE 18:**
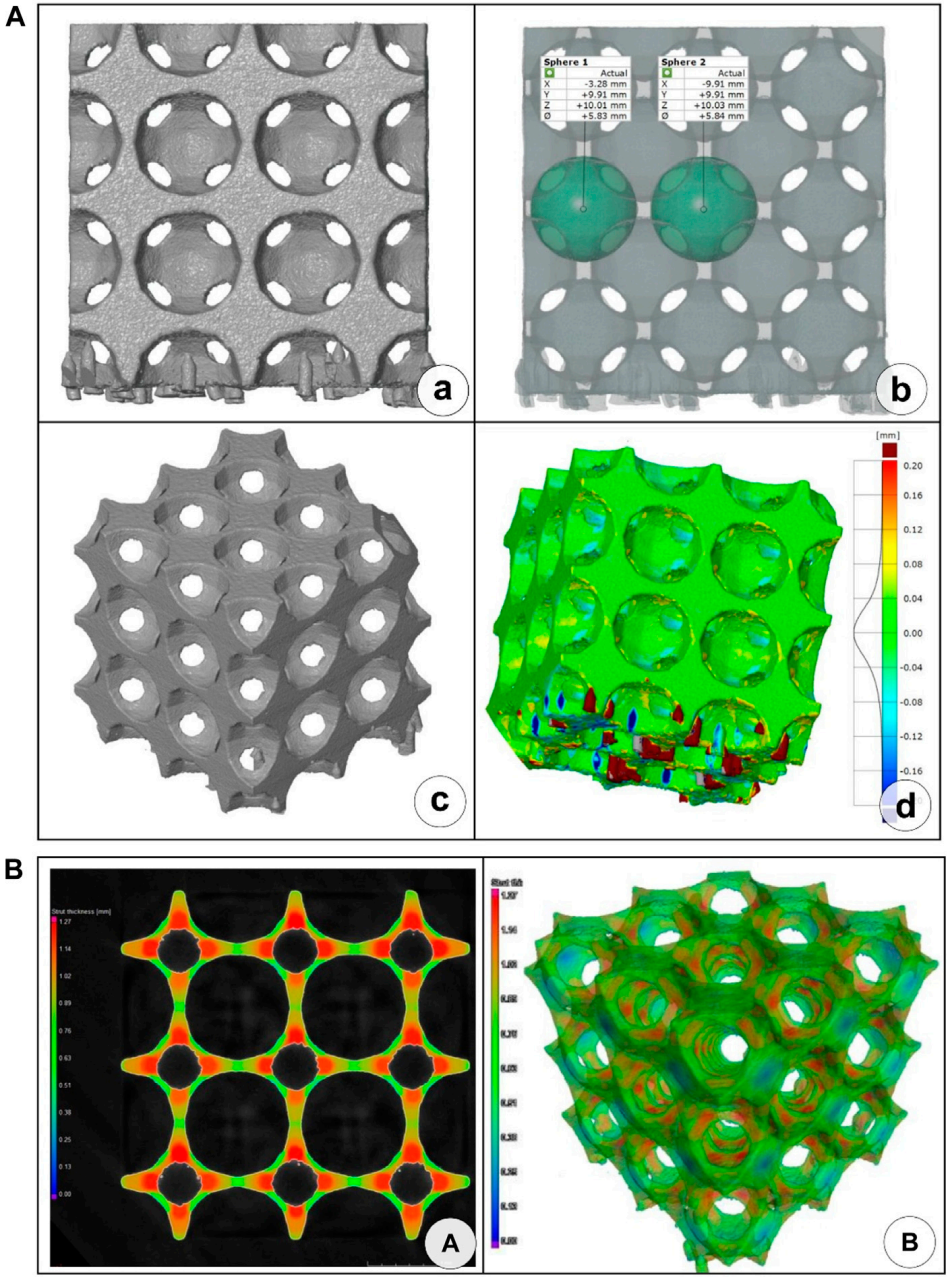
CT reconstruction of specimen no. 3. **(A)** front view of CT-reconstructed model; **(B)** measurement of cut-out sphere diameter; **(C)** axonometric view of CT-reconstructed model; **(D)** geometric mismatch between the 3D model and the printed specimens (calculated with Geometry software); **(E)** cell wall thickness dimensions in 2D section; **(F)** cell wall thickness dimensions in 3D reconstructed model.

The spherical diameters of all the three manufactured pieces were similarly smaller than their respective CAD counterpart designs. These size mismatches may have originated from the sand blasting that was utilized to remove the excess particles stuck on the surface after 3D printing (due to surface roughness). From the CT reconstructions, Figures 18A–D shows the results of specimen no. 3.

The geometric features of the lattice structure were also evaluated. These are also shown in Figures 18E–F for specimen no. 3.

## 5 Compression test results

The compression test results were compared to the finite element analysis outputs. The results from the first stage of the compression diagram were evaluated, as finite element analysis could calculate the elastic changes in the material structure. Thus, the comparisons were carried out until the maximum compression force. First, the compression test results are presented until the elastic limit, then the results of the finite element analysis are demonstrated.

The porosity values of the 3D models and the printed test specimens are presented in percentage format in Table 2.

**TABLE 2:** Porosity values.

	Porosity values of virtual 3D model (%)	Porosity values of printed specimen (%)
Specimen no. 1	75.7	70.7
Specimen no. 2	81.9	77.3
Specimen no. 3	84.7	80.7

**FIGURE 19:**
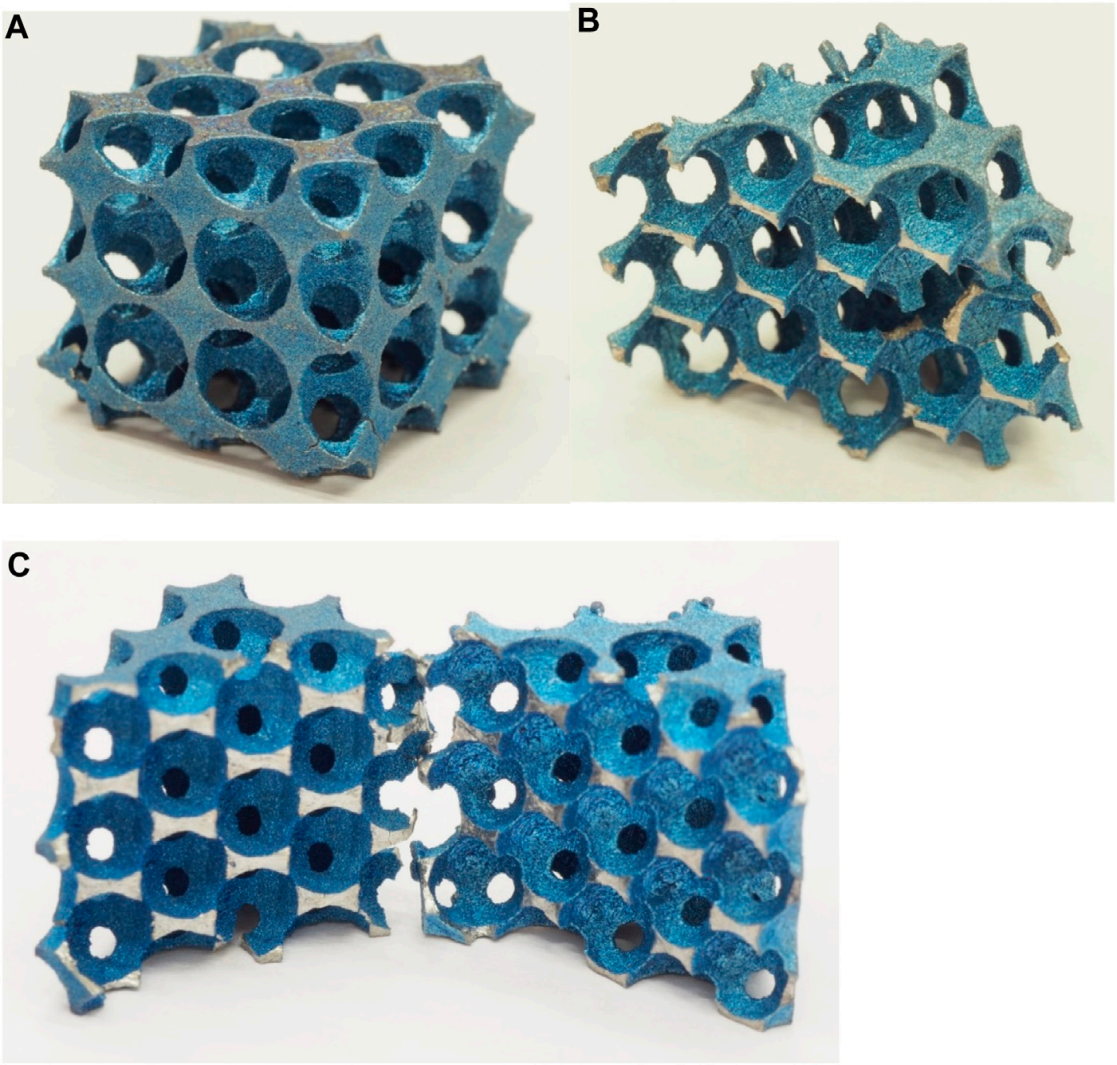
Photos of specimens: **(A)** Specimen no. 1 before the pressure test. **(B)** One broken half of Specimen no. 2. **(C)** The two broken pieces of Specimen no. 3.

The compression tests were performed according to the standard for cellular materials (DIN50134:2008). We used an Instron 5882 universal material testing machine with a load speed of 0.3 mm/s. The compression tests were executed on the different specimens. In order to reduce the costs of the titanium 3D printing, the specimens were prepared as a cube of (2 × 2 × 2 = 8) 8 unit cells, the edge length of the unit cube was 10 mm. Figure 19 shows the photos taken of the specimens before and after the compression test. The compression curves were calculated for the same specimens with finite element analysis.

Table 3 shows the results of the compressive tests conducted on the different specimen types.

**TABLE 3:** Compression test results of the three test specimens.

	Maximum compression force (N)	Compression at break (mm)
Specimen no. 1	63138.54	1.67
Specimen no. 2	41418.34	1.72
Specimen no. 3	33531.92	1.27

**FIGURE 20:**
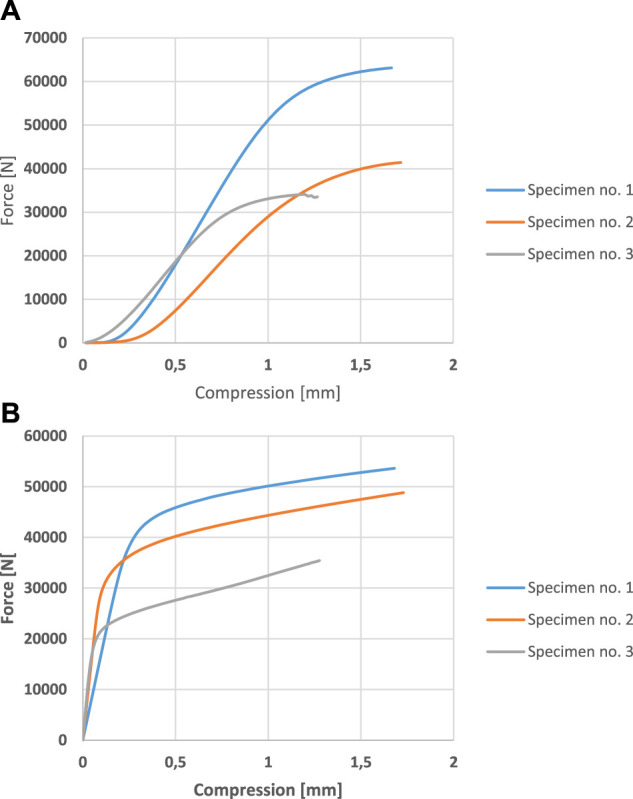
**(A)** Compression curves obtained from experimental setup. **(B)** Compression curves calculated with finite element analysis.

Figure 20 shows the measured (Figure 20A) and calculated (Figure 20B) compression curves in the elastic zone for the three different specimens.

It is noticeable in Figure 20A that a lower packing fraction i.e., a higher porosity, leads to a lower force at break. The maximum force difference was approximately 20000 N between test specimen no. 1 and no. 2 with no significant difference in compression length. Specimen no. 3 required a lower force to break, and fracture occurred earlier. The compressive tests showed the expected results.

The compression curves were calculated with finite element analysis as well for the three chosen models as shown in Figure 20B.

The results of the finite element analysis also provided the expected results. Figure 20B shows the lower force needed for break in structures with a higher porosity. Between specimen no.1 and no.2, the differences were smaller than in the measured diagrams, while the compression lengths were essentially identical. Specimen no.3 fractured earlier, and a substantially lower force was acting at break.

The pressure diagrams of the models calculated by the finite element simulation and those of the samples produced by the additive technology measured by real pressure tests have a similar course, but there are differences in the values. The magnitude of the differences in the compressive forces is acceptable and can be explained by the characteristics of the 3D printing. As shown above, there are small differences in the 3D printed samples compared to the pre-designed 3D model. There is a greater difference in compression between the calculated and measured results. At the initial stage, this can be explained partly by the slight slippage of the samples and partly by the nature of the intermittent measurement. Only compressive forces were considered in the validation of the finite element simulations.

The brittle fracture of the test specimen can be straightforwardly identified by analysing the compression curve. Brittle fracture is characteristic of Ti-6Al-4V titanium alloy, which was also proven by tensile tests presented in one of our previous studies (Kulcsár et al., 2018).

## 6 Conclusion

In this study, we investigated equivalent stress levels with respect to porosity in structures that were created by cut-out spheres distributed regularly considering simple cubic, face-centred cubic, body-centred cubic, and diamond structural alignments. The simple cubic structure resulted in the lowest possible volume reduction and the structure remained a closed-cell design, thus this solution was irrelevant. The other three structures all transitioned to open-cell lattices. The best results were achieved using body-centred cubic alignment, in which even high porosities came together with lower calculated mechanical stress rather than in the case of the face-centred cubic and the diamond lattice models. Thus, we investigated body-centred cubic structures further using 3D printed specimens, CT reconstruction, and compression tests. 3D printing resulted in minimal dimensional differences, which were caused by inadequate stress relief during the printing process and by particles that remained on the surface of the workpieces. As a result, the theoretical and experimental compressive curves showed minor differences. The finite element calculation results could be sufficiently validated due to the identical phases and limited differences in the curves.

## Data Availability

The original contributions presented in the study are included in the article/supplementary material, further inquiries can be directed to the corresponding author.
